# Esthetic preference of upper central incisor inclination in a smile profile view. A cross-sectional study

**DOI:** 10.4317/jced.62924

**Published:** 2025-08-01

**Authors:** Silvia Fuentes-Valera, Aron Aliaga Castillo, Fabián Reta-Martínez, Nicolás Arboleda-Ariza, Yalil Augusto Rodríguez-Cárdenas, Gustavo Armando Ruíz-Mora, Luis Ernesto Arriola-Guillén

**Affiliations:** 1 DDS. Orthodontic student, School of Dentistry, Universidad Científica del Sur, Lima, Perú; 2 DDS, MSc, PhD. Department of Orthodontics. University of Michigan, USA; 3 DDS, MSc. School of Dentistry, Universidad Científica del Sur, Lima, Perú; 4 DDS, MSc. Division of Oral and Maxillofacial Radiology, School of Dentistry, Universidad Científica del Sur, Lima, Perú; Division of Orthodontics, Faculty of Dentistry, Universidad El Bosque, Bogotá, Colombia; 5 DDS, MSc, PhD. Associate Professor of the Division of Oral and Maxillofacial Radiology, School of Dentistry, Universidad Científica del Sur, Lima, Perú. Occasional Professor of the Division of Orthodontics, Faculty of Dentistry, Universidad Nacional de Colombia, Bogotá D.C, Colombia; 6 DDS, MSc, PhD. Associate Professor of the Division of Orthodontics, Faculty of Dentistry, Universidad Nacional de Colombia, Bogotá D.C, Colombia; and Associate Professor of the Division of Oral and Maxillofacial Radiology, School of Dentistry, Universidad Científica del Sur, Lima, Perú; 7 DDS, MSc, PhD. Associate Professor of the Division of Orthodontics and Division of Oral and Maxillofacial Radiology, School of Dentistry, Universidad Científica del Sur, Lima, Perú

## Abstract

**Background:**

The objective of this study was to determine the esthetic preference of the upper central incisor (UCI) inclination from a smiling profile view in laypeople of Latin American origin.

**Material and Methods:**

This descriptive and cross-sectional study used a smiling profile photograph that was digitally modified to generate 7 types of UCI inclinations (+15°, +10°, +5°,0°, -10°, and -15°). Thus, 160 evaluators distributed in 4 groups (40 Peruvians, 39 Mexicans, 40 Brazilians and 41 Colombians) assessed the images using a visual analog scale. Kruskal-Wallis and Dunn Bonferroni tests were applied for multiple comparisons of ratings between groups (*p*<0.05).

**Results:**

The general sample of evaluators of the four nationalities considered -5° the most attractive slant and 0° the second most attractive slant, although a significant difference was found in the intensity of preference (*p*<0.001). Further, Peruvians rated the intensity of preference being -5° (Visual analogic scale (VAS) = 80 points) and 0° (VAS = 77 points) the most attractive inclinations, Mexicans rated 0° (VAS = 90 points) and -5°, -10° and 5° (VAS = 80 points) as the most attractive, Brazilians rated the most attractive inclinations at -5° and 0° (both with VAS = 80 points) and Colombians rated the most attractive inclinations at -5°, 0°, 5° and 10° (VAS = 60 points).

**Conclusions:**

Slightly retroclined (-5°) and neutral (0°) inclinations of central incisor were the most preferred by the Latin American individuals. This result should be considered by orthodontists for treatment planning.

** Key words:**Upper central incisors, inclination, esthetic preference, perception.

## Introduction

The search for facial beauty is one of the most important reasons why patients seek orthodontic treatment, as they have the desire to improve their appearance [1]. Nowadays, orthodontists not only aim to align teeth and restore function in the stomatognathic system, but they are also key agents in optimizing their patients’ facial attractiveness, [2,3] contributing to improve their self-perception and self-esteem [4,5].

Comprehensive evaluation of the smile is a very important element in facial esthetics and orthodontics, [6,7] which is why its analysis has been incorporated in a profile view in different specialties [8]. Currently, due to the fact that people spend a lot of time in their lives using the Internet, interacting in social networks, [9] media, in addition to the general habit of continuously taking “selfies” smiling in profile and videos exposing their image in different angles, patients are more rigorous and detailed in terms of their facial esthetic self-perception and that of others, taking great importance in the esthetics of the smile [10]. This is why when patients come to the office to improve their smile, they are no longer only concerned about their teeth being aligned; they have become more demanding and evaluate various elements of smile esthetics, among which is the inclination of the UCI [11-13].

Several studies have been aimed at establishing the ideal standard for evaluating UCI labiolingual positions and inclinations [14-16]. Several investigations have been conducted to find out the esthetic preference of UCI inclination in different parts of the world. Studies in West Asia [17-20] and South Asia [21-25] found that the preferred inclinations of laypeople were in the range of +5° to -5° and that they disliked excessive lingual and buccal inclination. Likewise, studies in East Asia, [26,27] found that the most pleasant inclination was -5°, while +15° was the least attractive. In the USA with a sample of diverse ethnic groups, including Caucasians and African Americans, it was observed that they preferred a more retro inclined inclination -8.3° and -8.8° respectively [28].

Previous research has shown that laypeople generally appreciate UCI inclination between +5° and -5° as pleasant, however, this should be evaluated in different sociocultural and geographical contexts, and there are no studies on this topic in Latin America, so it is necessary to know such preferences in Latin Americans, even though it is a large group, common aspects are usually present. Therefore, the purpose of this research was to determine the esthetic preference of Latin American adults on the UCI inclination in a smile profile view.

## Material and Methods

- Study Design

This cross-sectional descriptive study was reviewed by the Research Ethics Committee of Universidad Científica del Sur (approval number: POS-95-2024-00304). Besides, all evaluators provided informed consent to participate in the study.

- Study Setting

The study group consisted of 160 participants of both sexes, aged between 17 and 40 years, who were selected from orthodontic clinics in Lima (Peru), São Paulo (Brazil), Bogotá (Colombia), and Durango (Mexico). The participants included 40 Peruvians, 39 Mexicans, 40 Brazilians, and 41 Colombians. Inclusion criteria required individuals to be born and lifelong residents of their respective countries. Participants were excluded from the study if they had neurological disorders, cognitive disabilities, or any physical condition that would prevent them from completing the survey. Additionally, individuals who had lived in another country for more than one month, as well as students or professionals in dentistry, plastic surgery, or related fields of facial aesthetics, were not eligible for inclusion.

- Data Collection

A profile photograph of a 27-year-old female volunteer of Peruvian nationality was selected based on specific inclusion criteria. She exhibited a harmonious smile in both frontal and profile views and presented an orthognathic profile upon clinical examination. In lateral cephalometric analysis, she was classified with a Class I skeletal base (ANB = 2°) and had an upper central incisor inclination of 110° according to the UIPP (palatal plane - upper central incisor axis). Additionally, she demonstrated Class I dental relations with ideal overjet and overbite, with both the facial angle and H angle falling within the normal range, as described by Holdaway. Furthermore, her nasolabial angle and upper lip angle were also within the normal limits, as outlined by Arnett and Bergman [29].

A smiling photograph was taken of the face in profile on the right side, in the natural position of the corrected head, according to the Bass method [30] with the head placed by the operator in the “esthetic position” in which the face is not inclined; in such way the horizontal line (Hr’) was obtained, horizontal esthetic perpendicular to the true vertical that is not modified by the treatment. A photographic record was taken of the model with a social smile exposing the crown of the canine, using a Nikon D5100 reflex camera (Nikon, Japan), supported on a tripod. The camera was positioned at the height of the model’s head, oriented perpendicular to the true vertical line and parallel to the natural position of the head.

The patient was photographed standing 30 cm from a blue matte background wall and 1.5 m from the camera. The photograph was saved in RAW format and subsequently used for digital image alterations.

An expert professional was instructed in Photoshop CS6 version software to perform the alterations of the UCI inclinations following the guidelines described in previous studies [17,18,21,23,25]. The crown of the upper central incisor was cut out in the image editor program to generate the different inclinations, taking the incisal edge as the center of rotation (CRO). To maintain the vertical position of the UCI, a horizontal line was drawn as a tangent to the incisal edge.

**Figure 1:**
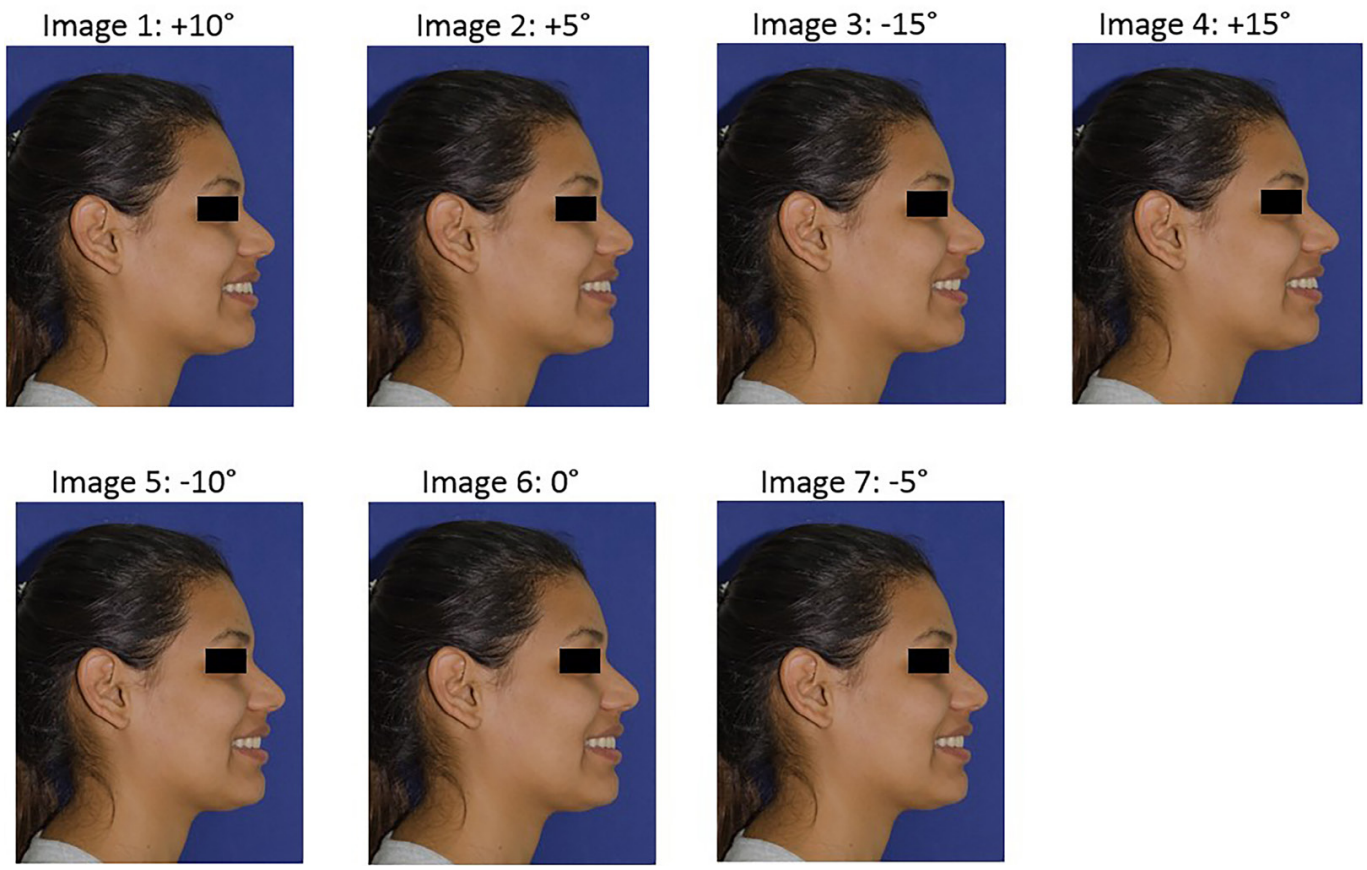
Normal UCI inclination and 6 modifications in labial and lingual direction, with 5° increments, were presented to the evaluators in random order.

**Figure 2:**
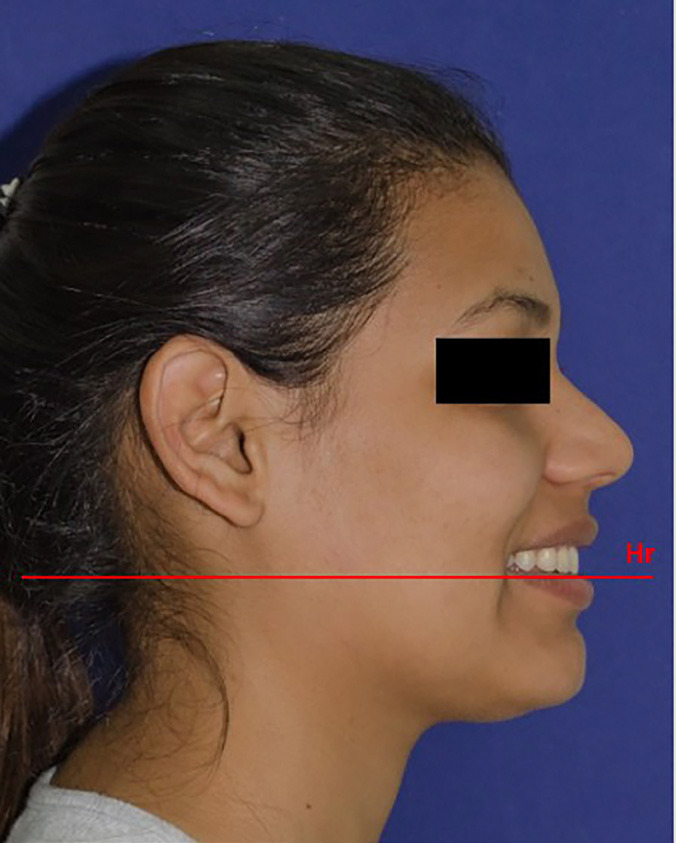
Original smiling profile image: Hr, true horizontal line, passing through the incisal edge of the UCI. The incisal edge of the UCI was taken as the center of rotation (CRO) to obtain the 6 modifications of the initial inclination of the incisor.

The initial inclination was taken as a reference to make the respective modifications of the inclination of the upper central incisor. A horizontal line was drawn tangent to the incisal edge to maintain its vertical position. In 5-degree increments, three modifications were made in the labial direction and three modifications in the palatal direction and artistic editing were performed as necessary to maintain a natural appearance. Seven final images (three labial, three palatal and one unaltered) were obtained (Figs. 1,2) and printed separately on photographic paper in a 13.7 x 17.4 cm format, creating a photo album, with one photograph per sheet.

The study had four collaborating dentists, who were instructed to give the same instructions to all the respondents in each country, the criteria for selection and exclusion of the participants to be chosen and the steps to be followed for the correct conduct of the survey. The instruction sheet for the collaborators, the photo album with instructions for the respondents and the cards for each respondent were mailed to each of them.

Each participant was given the photo album with the 7 randomly placed photographs giving them 10 minutes to solve the survey, the data collection document had a Visual Analog Scale (VAS) for each photograph, presented on a 100mm line numbered from 0 to 10, the extremes being 0 “very unpleasant” on the left and 10 “very pleasant” on the right. They were given no specific information about the images they would observe, except that the subject was female, and it was explained to them that they were to give a value to each photo based on the attractiveness of the model’s smile, marking with a vertical line, their evaluation on the 100-mm analog visual scale. They were asked not to return to the previously rated photographs as they progressed through the portfolio.

The exact scores were determined by measuring the position of the mark made by the individual on the visual scale, using a millimeter ruler for accuracy.

- Statistical analysis

The statistical program SPSS version 19 (Statistical Package for Social Sciences for Windows/ SPSS Inc., Chicago, IL, USA) with software for Windows was used for data processing. Baseline characteristics of the sample were compared between groups using ANOVA, HSD Tukey (age) and chi-square (sex) tests. Descriptive statistics (mean and standard deviation) were performed for a rater age according to nationality, while median and quartiles Q1 and Q3 were calculated for VAS ratings, for each UCI inclination category, for each nationality. Subsequently, the Kruskall Wallis nonparametric test and the Dunn Bonferroni multiple comparisons analysis were used to compare the VAS scores among the 4 groups (nationality). We worked at a significance level of *p*<0.05 for all tests.

## Results

The initial characteristics of the sample are shown in  Table 1, with the sex distribution being similar in the evaluators from the four participating countries (*p*=0.789). Likewise, age showed a significant difference mainly with lower age in the Colombian group (20.68 ± 4.56) with respect to the other three groups (*p*>0.001).

 Table 2 shows the comparison of the esthetic smile preference (VAS) for 7 different UCI inclinations according to each nationality. In general, the evaluated sample of the four nationalities considered the most attractive inclination to be -5° and in second place 0°, although a significant difference was found in the intensity (*p*<0.001), all pointed out these two inclinations as the attractive ones. Specifically, Peruvians rated -5° (VAS = 80) and 0° (VAS = 77) as the most attractive inclinations, Mexicans rated 0° (VAS =90) and -5°, -10° and 5° (VAS=80 points) as the most attractive. Brazilians rated the most attractive inclinations at -5° and 0° (both with VAS = 80) and Colombians rated the most attractive inclinations at -5°, 0°, 5° and 10° (VAS = 60).

## Discussion

The value of aesthetic preference regarding UCI inclination varies between studies and is often classified as pleasant, acceptable, or unpleasant. Some authors considered the inclination of -10° to be aesthetically unpleasant, [18,24,25] while others found that values of +15° [17,18-21,23,26] and -15° [17] were rated as very unpleasant. In general, in literature it seems that there is a lower esthetic perception on inclinations equal to or greater than 10°, both palatal and labial; however, not all studies agree on which value is the most pleasant and this result could be modified by racial and environmental factors. For this reason, the present investigation sought to evaluate the esthetic preference of Latin American adults on the inclination of the UCI in a smile profile view.

The esthetic preference of UCI inclination found in the Latin American sample of our study was in favor of slightly retroclined (-5°) or upright/neutral (0°) incisor inclinations, results that agree with several investigations carried out in laypeople of other racial groups and localities such as in South Asian [23-25] or West Asian individuals [18-20]. This same trend was found in dental students in studies conducted in China [26] and India [22]. On the contrary, very few studies such as Ghaleb *et al*. [17] reported that the most pleasing inclination of the incisors was +5°, that is, labially inclined. This being so, it can be noted that in general the preference of UCI inclination globally seems to be the same and seems to go beyond the racial factor or the geographical environment of the individuals.

On the other hand, when comparing whether our study presented any differences between individuals of different nationalities, we observed that in general our Latin American respondents had similar esthetic preferences, preferring mainly a retroclined UCI inclination (-5°) and in some specific cases upright/ neutral (0°), but very close to the previous value. Therefore, these common results among them could be taken into consideration by orthodontists in their treatment planning so that they can obtain satisfactory results with respect to what their patients are likely to desire.

When comparing the least preferred inclinations in our study, we found a similar response, only the Mexican, Brazilian, and Colombian groups rated very proclined incisors (+15°) the lowest, and the Peruvian group rated +10° the lowest. These findings coincided with several studies that also found that severe labial inclinations (+15°) were rated as the most unpleasant [17,19,21,23,26]. Therefore, it is advisable for clinicians to consider these findings specifically in procedures that include dentoalveolar expansion for camouflage orthodontic treatments.

One of the limitations of our study was the use of full-face photographs as they could have generated distractions in the evaluators, however, it was decided to replicate the protocol described in previous articles and thus be able to compare the results. Another aspect to consider in our study was the age of the evaluators, which was similar among them; however, the group of Colombian evaluators was slightly younger and although it had significant differences with respect to the other groups, the tendency of their preference did not vary from the other groups, although their ratings were lower in general.

Finally, the results obtained in this research can be used by orthodontists in the different countries evaluated since it is important to consider the opinion of potential patients who probably consider the same preferences as the evaluators participating in this study regarding the inclination of the incisors and thus can develop treatment plans according to the esthetic requirements of their patients.

## Conclusions

Slightly negative (-5°) and neutral (0°) upper central incisor inclinations were the inclinations that lay Latin American individuals preferred the most. This result should be considered by orthodontists when planning their treatments.

## Figures and Tables

**Table 1:** Table Sociodemographic characteristics of the evaluators according to nationality.

Sociodemographic characteristics		Peruvian (n=40)	Mexican (n=39)	Brazilian (n=40)	Colombian (n=41)	p-value
Sex	Female	21 (24.4%)	19 (22.1%)	24 (27.9%)	22 (25.6%)	0.789*
	Male	19 (25.7%)	20 (27.0%)	16 (21.6%)	19 (25.7%)	
Age	(Mean ± SD)	28.65 ± 6.75a	30.92 ± 5.88a	31.15 ± 6.52a	20.68 ± 4.56b	<0.001**

* Chi-square test; ** ANOVA; *p*<0.05; different letters indicate significant differences (HSD Tukey test).

**Table 2:** Table Facial attractiveness ratings by upper central incisor inclinations by panels of raters.

		-15°	-10°	-5°	0°	+5°	+10°	+15°
Nationality	n (%)	Me (Q1 -Q3)	Me (Q1 -Q3)	Me (Q1 -Q3)	Me (Q1 -Q3)	Me (Q1 -Q3)	Me (Q1 -Q3)	Me (Q1 -Q3)
Peruvian	40	50.0 (32.5 - 65.75) a	57.5 (45.75 - 74.25) a	80.0 (70.0 - 86.5)b	77.0 (70.0 - 85.0)b	60.0 (50.0 - 70.0)ab	47.0 (40.0 - 59.5)a	50.0 (40.0 - 59.25)a
Mexican	39	80.0 (60.0 - 80.0) b	80.0 (70.0 - 90.0) b	80.0 (70.0 - 90.0)b	90.0 (80.0 - 90.0)c	80.0 (70.0 - 90.0)c	70.0 (60.0 - 90.0)c	70.0 (60.0 - 90.0)b
Brazilian	40	70.0 (50.0 - 80.0) b	70.0 (60.0 - 90.0) b	80.0 (70.0 - 100.0)b	80.0 (60.0 - 100.0)bc	70.0 (50.0 - 80.0)bc	70.0 (50.0 - 80.0)bc	60.0 (50.0 - 80.0)b
Colombian	41	50.0 (40.0 - 60.0) a	50.0 (40.0 - 70.0) a	60.0 (50.0 -77.5)a	60.0 (50.0 - 72.5)a	60.0 (45.0 - 70.0)a	60.0 (40.0 - 70.0)ab	45.0 (38.0 - 67.5)a
p_value*		<0.001	<0.001	<0.001	<0.001	<0.001	<0.001	<0.001

* Kruskal Wallis test; *p*<0.05; different letters indicate significant differences (Dunn Bonferroni test). Me: median; Q1: first quartile; Q3: third quartile

## Data Availability

The datasets used and/or analyzed during the current study are available from the corresponding author.
